# Right-sided Bochdalek hernia in an adult: a case report

**DOI:** 10.1186/1752-1947-3-9291

**Published:** 2009-11-23

**Authors:** Elina Laaksonen, Seppo Silvasti, Tapio Hakala

**Affiliations:** 1 Department of Surgery, North Karelia Hospital, Joensuu, Finland

## Abstract

**Introduction:**

Bochdalek hernia is a type of congenital diaphragmatic hernia that typically presents in childhood - the clinical manifestation of symptoms and diagnosis in adults is extremely rare. There are fewer than 20 cases of right-sided Bochdalek hernia reported in adults in the literature.

**Case presentation:**

We review a case of a 38-year-old woman with a right-sided Bochdalek hernia who was experiencing abdominal pain and pleural effusion. The diagnosis of Bochdalek hernia was made by chest X-ray and computed tomography. The right lobe of the liver and flexura hepatica of the colon were herniated into the thorax cavity. The hernia was treated via thoracotomy assisted with laparoscopy and she made an uneventful recovery.

**Conclusion:**

We report a rare case of a right-sided Bochdalek hernia for which our patient was treated successfully. Even though rare, this disorder should be recognised, examined and treated appropriately to avoid complications.

## Introduction

In 1848, Bochdalek first described a congenital hernia resulting from the developmental failure of the posterolateral foramina to fuse properly [1]. Bochdalek hernia is a congenital anomaly normally diagnosed in neonatal and postnatal patients - the clinical manifestation of symptoms and diagnosis in adults is extremely rare. Most of the hernias (80 to 90%) are found on the left side. Right-sided hernias are rarer because the right pleuroperitoneal canal closes earlier and the liver buttresses the right diaphragm [2]. There are fewer than 100 cases of Bochdalek hernia reported in adults in the literature and fewer than 20 of those cases involve right-sided hernias [1].

We report the case of a 38-year-old woman whose right-sided Bochdalek hernia was treated via thoracotomy assisted with laparoscopy.

## Case presentation

A 38-year-old nurse presented with complaints of a history of abdominal pain and nausea. She had been diagnosed with endometriosis and her left ovary had been removed a few years earlier. She was taking no medication and had no other underlying disease. There was no history of any previous abdominal or thoracic trauma. She had given birth 9 months earlier.

**Figure 1:**
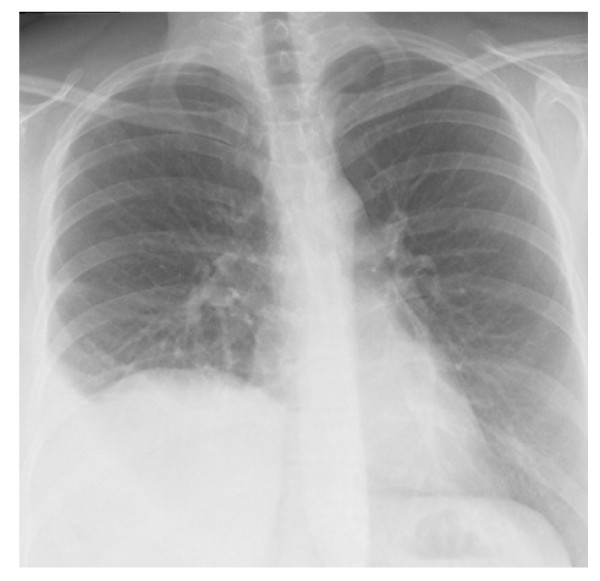
**Preoperative chest X-ray showing a right-sided pleural effusion and an elevated right hemidiaphragm**.

**Figure 2:**
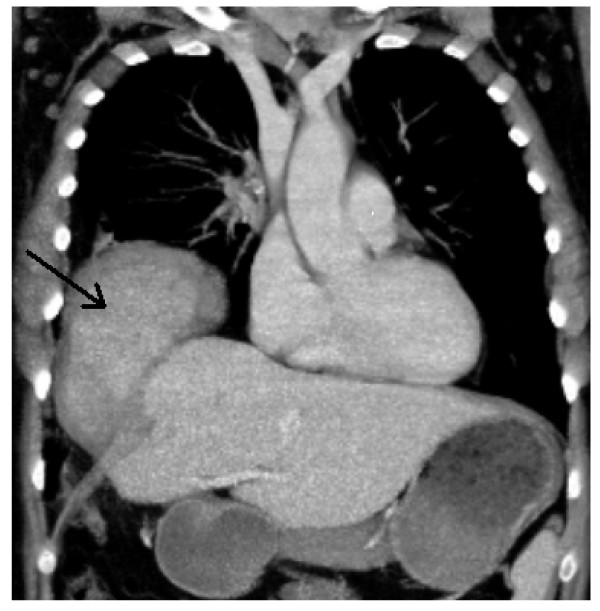
**Computed tomography of the thorax and abdomen**. At the sagittal section, a black arrow indicates the right lobe of the liver in the thorax cavity.

Physical examination revealed pain on the epigastric area and on the left-lower abdomen. Gynecological examination and ultrasound of the abdomen revealed no abnormal findings. A chest X-ray revealed that the right diaphragm was unexceptionally high and there was also a pleural effusion present on the right side (Figure 1). A computed tomography (CT) scan was performed and confirmed the diagnosis as a right-sided Bochdalek hernia (Figure 2).

Our patient underwent an elective operation and, initially, laparoscopy was performed. There was no hernia sac present, and the right lobe of the liver, flexura hepatica of the colon and omentum were herniated into the thorax cavity. No signs of endometriosis were detected on the surface of the diaphragm. After the laparoscopy, short lateral thoracotomy into the sixth intercostal space was performed. The herniated organs were moved back to the abdominal cavity. A 10-cm wide defect in the diaphragm was closed with interrupted sutures, and a resorbable patch (Dexon, Covidien, CT, USA) was sutured under the diaphragm to reinforce the defect.

The postoperative course was uneventful and the patient was discharged 7 days after the operation. At follow-up, she had no symptoms and her chest X-ray was normal.

## Discussion

Bochdalek hernia is a congenital anomaly in neonatal and postnatal patients and occurs in about one in 2,200 to 12,500 live births, but it is rare in adults [3]. Most Bochdalek hernias are diagnosed in children who present with acute pulmonary symptoms [4]. In contrast to the acute presentation by infants with these hernias, most adults present with more chronic symptoms, such as chronic dyspnoea, chest pain and pleural effusion. Recurrent abdominal pain, postprandial fullness and vomiting are the most common abdominal symptoms in adults [2]. Our patient was experiencing the typical symptoms of abdominal pain and pleural effusion. Some patients have no symptoms and the disorder is unexpectedly detected on chest X-ray [5,6]. The hernia size varies and the content of the hernial sac may differ in each case. In 50% of acute presentations, the hernia sac contains colon, and in 40% the sac may contain multiple other viscera including small bowel, stomach, liver, kidney and gallbladder [1]. The clinical presentation of a right-sided Bochdalek hernia can also manifest as strangulation of the contents of the hernia, colon necrosis and hemothorax [4,5]. A Bochdalek hernia can also masquerade as a tension pneumothorax on the chest X-ray, which can complicate the treatment [7].

Increased intra-abdominal pressure of long duration, such as that seen during pregnancy, and also the acute rise in pressure during parturition, can predispose the mother to herniation [2]. In our case the patient had given birth 9 months earlier. A hernia sac has been reported to be present in 10 to 38% of such cases [4]. Long-term survival may be due to the persistence of a pleuroperitoneal sac, and the rupture of the sac in adult life may trigger the characteristic symptoms [4]. In our patient, no hernia sac was present.

The diagnosis in our patient was ascertained by a combination of chest X-ray and CT. On chest X-ray, a Bochdalek hernia can show up as gas and fluid-filled viscera or, as in our patient, as a pleural effusion. Contrast-enhanced CT is the most useful examination for this diagnosis. Typical findings are fat or soft tissue contour on the upper surface of the diaphragm. Another characteristic of a Bochdalek hernia is its posterolateral location. These findings were also present in our patient. Other possible investigations include upper gastrointestinal contrast series, which can exclude malrotation but may miss complications [1]. Small defects of the diaphragm caused by endometriosis have been described in the literature [8]. Usually these defects appear on the centrum tendineum of the diaphragm and endometrial implants may also be present. In addition, in the reported cases of endometrial lesions and perforations on the diaphragm, the patients have presented with signs of a pneumothorax [8]. Because our patient had no signs of endometriosis on the diaphragm, no pneumothorax and the location of the diaphragmatic defect was posterolateral, our diagnosis was a Bochdalek hernia. A chest X-ray taken ten years earlier revealed that the right diaphragm was already exceptionally high.

Management of a Bochdalek hernia includes reducing the abdominal contents and repairing the defect through a laparotomy or thoracotomy. Successful laparoscopic and thoracoscopic repairs of Bochdalek hernias have both been described. Right-sided defects are traditionally dealt with by a thoracic or thoracoabdominal approach because of the presence of the liver. For left-sided hernias some advocate a transthoracic approach while others suggest a transperitoneal approach [1,3]. In our patient the size of the defect in the diaphragm that we detected during the laparoscopy was 10 cm wide. Because of the large size of the defect we decided to perform a thoracotomy and open repair of the hernia instead of attempting thoracoscopic repair.

## Conclusion

We report a rare case of a right-sided Bochdalek hernia in an adult who was treated via thoracotomy and laparoscopy. People with a Bochdalek hernia may not have any symptoms and the disorder may be detected unexpectedly, or the symptoms and expressions may vary from mild to serious complications. Even though rare, this disorder should be recognised, examined and treated appropriately to avoid complications.

## Abbreviations

CT: computed tomography.

## Consent

Written informed consent was obtained from the patient for publication of this case report and accompanying images. A copy of the written consent is available for review by the Editor-in-Chief of this journal.

## Competing interests

The authors declare that they have no competing interests.

## Authors' contributions

EL was a major contributor in writing the manuscript. TH together with SS operated on the patient and revised the manuscript. All authors read and approved the final manuscript.

## References

[B1] Rout S Foo FJ Hayden JD Guthrie A Smith AM Right-sided Bochdalek hernia obstructing in an adult: case report and review of the literature Hernia 2007 11 359 362 10.1007/s10029-007-0188-5 17342385

[B2] Kanazawa A Yoshioka Y Inoi O Murase J Kinoshita H Acute respiratory failure caused by an incarcerated right-sided adult Bochdalek hernia: report of a case Surg Today 2002 32 812 815 10.1007/s005950200156 12203061

[B3] Yamaguchi M Kuwano H Hashizume M Sugio K Sugimachi K Hyoudou Y Thoracoscopic treatment of Bochdalek hernia in the adult: report of a case Ann Thorac Cardiovasc Surg 2002 8 106 108 12027798

[B4] Kocakusak A Arikan S Senturk O Ycel AF Bochdalek's hernia in an adult with colon necrosis Hernia 2005 9 284 287 10.1007/s10029-004-0302-x 16450080

[B5] Niwa T Nakamura A Kato T Kutsuna T Tonegawa K Kawai A Itoh M An adult case of Bochdalek hernia complicated with hemothorax Respiration 2003 70 644 646 10.1159/000075213 14732798

[B6] Shin MS Mullign SA Baxley WA Ho KJ Bochdalek hernia of diaphragm in the adult. Diagnosis by computer tomography Chest 1987 92 1098 1101 10.1378/chest.92.6.1098 3677819

[B7] Dalton AM Hodgson RS Crossley C Bochdalek hernia masquerading as a tension pneumothorax Emerg Med J 2004 21 393 394 10.1136/emj.2002.004697 15107395 PMC1726336

[B8] Korom S Canyurt H Missbach A Schneiter D Kurrer MO Haller U Keller PJ Furrer M Weder W Catamenial pneumothorax revisited: clinical approach and systemic review of the literature J Thorac Cardiovasc Surg 2004 128 4 502 508 10.1016/S0022-5223(04)00772-X 15457149

